# Virulence of *Mycobacterium intracellulare* clinical strains in a mouse model of lung infection – role of neutrophilic inflammation in disease severity

**DOI:** 10.1186/s12866-023-02831-y

**Published:** 2023-04-03

**Authors:** Yoshitaka Tateishi, Yuriko Ozeki, Akihito Nishiyama, Mari Miki, Ryoji Maekura, Hiroshi Kida, Sohkichi Matsumoto

**Affiliations:** 1 Department of Bacteriology, Graduate School of Medical and Dental Sciences, Niigata University, 1-757, Asahimachi-Dori, Chuo-Ku, Niigata, 951-8510 Japan; 2 Tokushima Prefecture Naruto Hospital, Tokushima, Japan; 3 Department of Respiratory Medicine, National Hospital Organization Osaka Toneyama Medical Center, Toyonaka, Japan; 4 Laboratory of Tuberculosis, Institute of Tropical Disease, Universitas Airlangga, Surabaya, East Java, Indonesia

**Keywords:** Nontuberculous mycobacteria, *Mycobacterium intracellulare*, Virulence, Neutrophil, Chemotherapeutic efficacy

## Abstract

**Background:**

*Mycobacterium intracellulare* is a major etiological agent of *Mycobacterium avium-intracellulare* pulmonary disease (MAC-PD). However, the characteristics of the virulence of *M. intracellulare* and the in vivo chemotherapeutic efficacy remain unclear. In this study, we examined the virulence of nine *M. intracellulare* strains with different clinical phenotypes and genotypes in C57BL/6 mice.

**Results:**

We classified three types of virulence phenotypes (high, intermediate, and low) based on the kinetics of the bacterial load, histological lung inflammation, and neutrophilic infiltration. High virulence strains showed more severe neutrophilic infiltration in the lungs than intermediate and low virulence strains, with 6.27-fold and 11.0-fold differences of the average percentage of neutrophils in bronchoalveolar lavage fluid, respectively. In particular, the high virulence strain M.i.198 showed the highest mortality in mice, which corresponded to the rapid progression of clinical disease. In mice infected with the drug-sensitive high virulence strain M019, clarithromycin-containing chemotherapy showed the highest efficacy. Monotherapy with rifampicin exacerbated lung inflammation with increased lymphocytic and neutrophilic infiltration into the lungs.

**Conclusions:**

The virulence phenotypes of clinical strains of *M. intracellulare* were diverse, with high virulence strains being associated with neutrophilic infiltration and disease progression in infected mice. These high virulence strains were proposed as a useful subject for in vivo chemotherapeutic experiments.

**Supplementary Information:**

The online version contains supplementary material available at 10.1186/s12866-023-02831-y.

## Introduction

In contrast to the decline in tuberculosis prevalence in developed countries, the prevalence of nontuberculous mycobacterial diseases is increasing worldwide, including in East Asia, Europe, and the United States [1]. In the majority of cases, nontuberculous mycobacterial disease presents as *Mycobacterium avium-intracellulare* pulmonary disease (MAC-PD) [2]. In East Asia, including Japan, *M. intracellulare* is the second major causative agent of MAC-PD after *M. avium* [3]. In contrast to tuberculosis, MAC-PD is difficult to cure even using long-term multidrug chemotherapy, including new macrolides [4, 5]. Several studies have reported that 15% of patients with extensive pulmonary cavitary lesions die after repeated relapses and exacerbations [6]. To improve the prognosis of MAC-PD, it is necessary to elucidate the pathogenicity of bacteria that affect disease progression and to establish a system to evaluate the efficacy of in vivo chemotherapy.

The pathogenesis of MAC-PD is considered in terms of the balance between the host immune response and bacterial virulence. One host factor suggested to be involved is a defective interferon-gamma (IFN-γ) response, as evidenced by disseminated MAC disease in cases possessing a genetic mutation of the IFN-γ receptor [7, 8] or autoantibodies to IFN-γ in non- acquired immunodeficiency disease syndrome (AIDS) patients [9, 10]. In addition, the inhibitory cytokine interleukin-10 (IL-10) is suggested to increase susceptibility in mouse experiments [11]. Bacterial virulence factors have also been suggested to influence the pathogenesis of MAC-PD, with specific serovars contributing to the clinical severity of disease. In some studies, virulence was compared between MAC strains isolated from the environment and those isolated from infected human patients and animals [12]. Serovar 4 of *M. avium* has been associated with disease severity in AIDS and non-AIDS MAC disease patients [13, 14]. We previously examined the experimental virulence of six *M. avium* and two *M. intracellulare* strains in C57BL/6 mice and found that the hypervirulent strain M.i.198 persisted in monocyte-derived macrophages, caused a high bacterial load, and resulted in severe lung inflammation and serious clinical disease outcomes [15]. Since *M. intracellulare* is suggested to cause more severe clinical disease progression than *M. avium* [16], increased knowledge on the virulence of *M. intracellulare* is crucial. However, to date, few studies have characterized the virulence phenotypes of the increasing number of clinical *M. intracellulare* strains or the impact of virulence on experimental outcomes, including the lung inflammation pattern and mortality rates.

Comparative genomic analyses have been conducted extensively in *M. avium* and *M. intracellulare* [17–19]. As a common finding, MAC is genetically diverse, unlike *Mycobacterium tuberculosis*. *M. avium* can be classified into several subspecies based on genomic diversity, including subspecies *avium, hominissuis, paratuberculosis,* and *silvaticum* [17]. In *M. avium* subspecies *hominissuis*, strains belonging to some clusters have been suggested to possess potential genetic factors that account for their clinical pathogenesis [18]. We have demonstrated two main genotypes of *M. intracellulare*: typical *M. intracellulare* (TMI) and *M. paraintracellulare-M. indicus pranii* (MP-MIP) (Additional file 1) [19]. However, comprehensive evidence of the relationship between specific genotypes and the virulence phenotypes has not been established.

To evaluate the efficacy of novel chemotherapeutic drugs in vivo, it is necessary to determine the optimal strains that exhibit virulence in the infected organs of mice. For MAC-PD, consistency between routine susceptibility testing of MAC isolates and clinical susceptibility has only been established for clarithromycin (CAM) [4, 5]. No standard method of susceptibility testing has been established for any other chemotherapeutic drugs. Therefore, to establish an evaluation system for chemotherapeutic efficacy it is necessary to determine the effect of each drug on bactericidal activity and on inflammation repair and also to compare the chemotherapeutic efficacy of novel drugs with pre-existing drugs.

In this study, we performed an infection experiment using *M. intracellulare* strains with different genotypes and clinical manifestations to elucidate the relationship between differences in virulence and clinical disease progression. Using virulent strains, we established a mouse model of infection for evaluating in vivo chemotherapeutic efficacies in MAC-PD.

## Results

### Clinical phenotypes of *M. intracellulare* strains

The strains used in this study are listed in Table 1 and Additional file 2. M.i.198, M.i.27 and M018 belonged to the TMI genotype (Additional file 1). M001, M003, M019 and M021 belonged to the MP-MIP genotype. Type strains of the TMI genotype (ATCC13950) and MP-MIP genotype (MOTT64) were also included in the study.

**Table 1:** Characteristics of *M. intracellulare* strains used in this study

Strain	Genotype^a^	Clinical phenotype	Bacterial burden and lung inflammation in C57BL/6 mice	Histological feature
ATCC13950	TMI	Type strain of TMI genotype	Low	Almost no inflammation throughout infection
M.i.198	TMI	Progressive	High	Peribronchovascular and alveolar inflammation
M.i.27	TMI	Stable	Intermediate	Peribronchovascular and alveolar inflammation
M018	TMI	Stable	Intermediate	Peribronchovascular and alveolar inflammation
MOTT64	MP-MIP	Type strain of MP-MIP genotype	Low	Lung inflammation resolved after 8 weeks of infection
M001	MP-MIP	Progressive	Intermediate	Peribronchovascular and alveolar inflammation
M003	MP-MIP	Progressive	Low	Lung inflammation resolved after 8 weeks of infection
M019	MP-MIP	Stable	High	Peribronchovascular and alveolar inflammation
M021	MP-MIP	Stable	High	Peribronchovascular and alveolar inflammation

^a^*TMI* Typical *Mycobacterium intracellulare* genotype, *MP-MIP Mycobacterium paraintracellulare-M. indicus pranii* genotype

Clinical phenotypes were classified based on chest X-ray findings and laboratory tests including the erythrocyte sedimentation ratio and the number of sputum bacteria [20]. M.i.198, M001 and M003 were isolated from patients with clinically progressive disease characterized by the large number of bacteria in sputum, extensive lesions with cavities and consolidations, and high minimum inhibitory concentration (MIC) value of clarithromycin (CAM) in relation to the poor responsiveness to chemotherapy. M.i.27, M018, M019 and M021 were isolated from patients with clinically stable disease characterized by the small number of bacteria in sputum, limited lesions with less or no cavities, and low MIC value of clarithromycin (CAM). M018 and M021 were isolated from patients whose disease was able to be controlled by CAM-containing chemotherapy. M.i.27 and M019 were isolated from patients whose disease was able to be controlled without chemotherapy. Except for M.i.27, M019 and M021, the sampling of sputum was performed during chemotherapy.

### Kinetics of the bacterial load

**Fig. 1:**
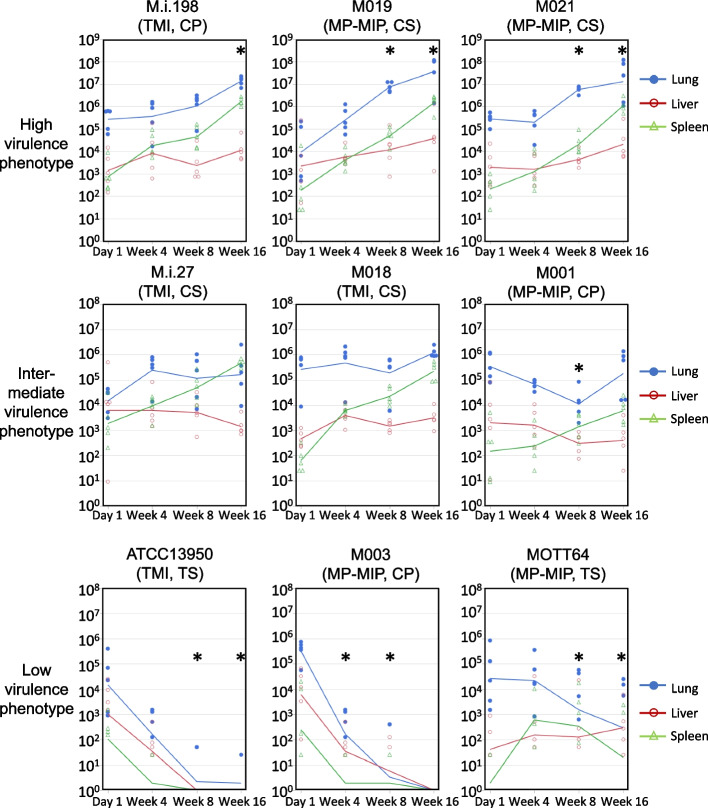
Kinetics of bacterial growth in the lungs, liver, and spleen of C57BL/6 mice infected with *M. intracellulare* strains. The lungs, liver, and spleen of five mice were sectioned per strain at each time-point. Each point represents CFUs per organ from a single animal. Lines between timepoints indicate the moving average. Blue: lungs, Red: liver, Green: spleen. No colonies were recovered in some samples of low virulence strains (Additional file 3 in detail). *: The CFUs in the lungs were statistically significantly different compared with those at day 1 after infection. TMI: typical *M. intracellulare* genotype, MP-MIP: *M. paraintracellulare-M. indicus pranii* genotype, CP: clinically progressive cases, CS: clinically stable cases, TS: type strain

Since mycobacteria do not produce exotoxins, bacterial replication is essential to induce active mycobacterial disease such as induction of lung inflammation and granulomatous necrotic lesions [21]. Therefore, to evaluate the virulence of each *M. intracellulare* strains, we used bacterial burden and induction of lung inflammation in mice as an objective index of virulence of mycobacteria. To evaluate the virulence of each strain, 1 × 10^6^ colony forming units (CFUs) of bacteria were instilled intratracheally, and the bacterial loads in the lung, liver, and spleen were calculated. The kinetics of the bacterial load in the lungs (between day 1 and 16 weeks of infection) could be classified into three virulence phenotypes (Fig. 1, Additional file 3). M.i.198, M019, and M021 showed a high virulence phenotype with logarithmically increasing bacterial loads in the lungs. M.i.27, M018, and M001 showed an intermediate virulence phenotype with chronic infection at a constant bacterial load in the lungs. ATCC13950, M003, and MOTT64 showed a low virulence phenotype with the constant decrease in the bacterial load in the lungs (Additional file 3). For M.i.198 (a high virulence strain), the kinetics of the bacterial load and histological lung inflammation in mice was in accordance with the rapid progression of clinical disease [15]. However, the virulence in mice infected with other strains did not necessarily correspond to the genotypes and clinical phenotypes. This was because the bacterial load in the lungs was not increased in strains isolated from clinically progressive cases such as M001 (an intermediate virulence strain) and M003 (a low virulence strain), whereas the bacterial load in the lungs was continuously increased in strains isolated from clinically stable cases such as M019 and M021 (high virulence strains).

**Fig. 2:**
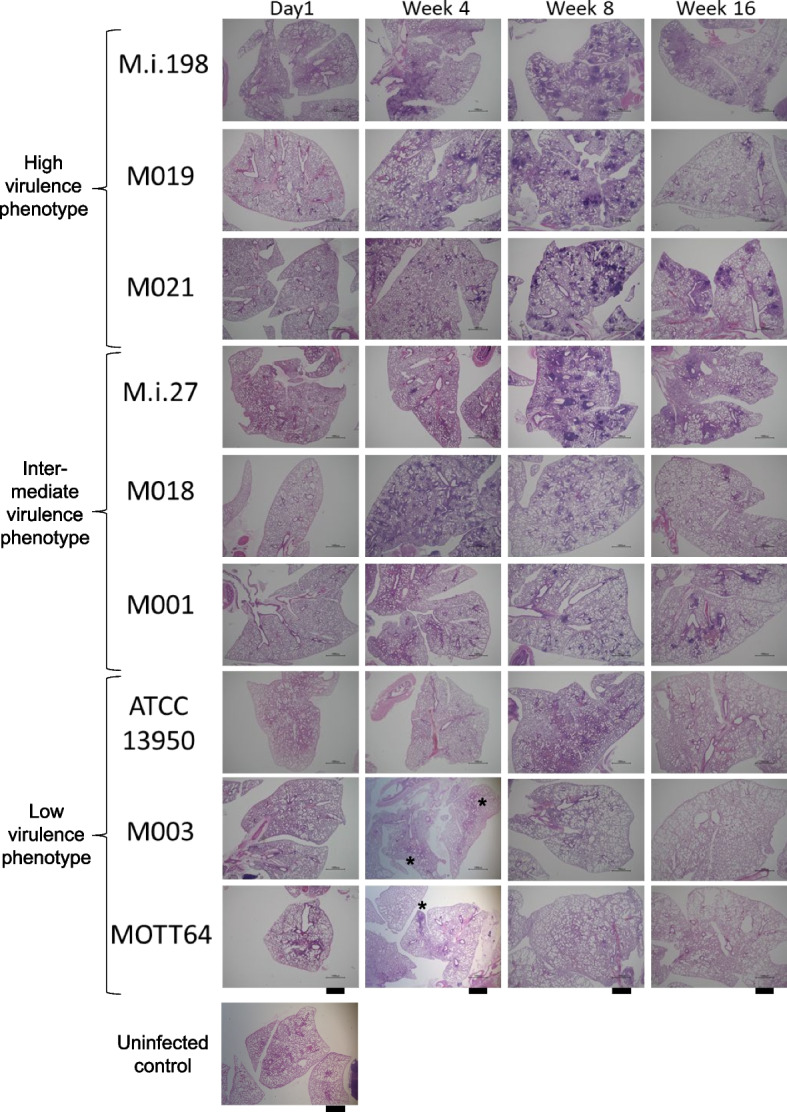
Histological images of the lungs during 16 weeks of infection in C57BL/6 mice by hematoxylin–eosin staining. Bars indicate 1 mm. Histological lung inflammation at 4 weeks of infection with M003 and MOTT64 was indicated by asterisks

**Fig. 3:**
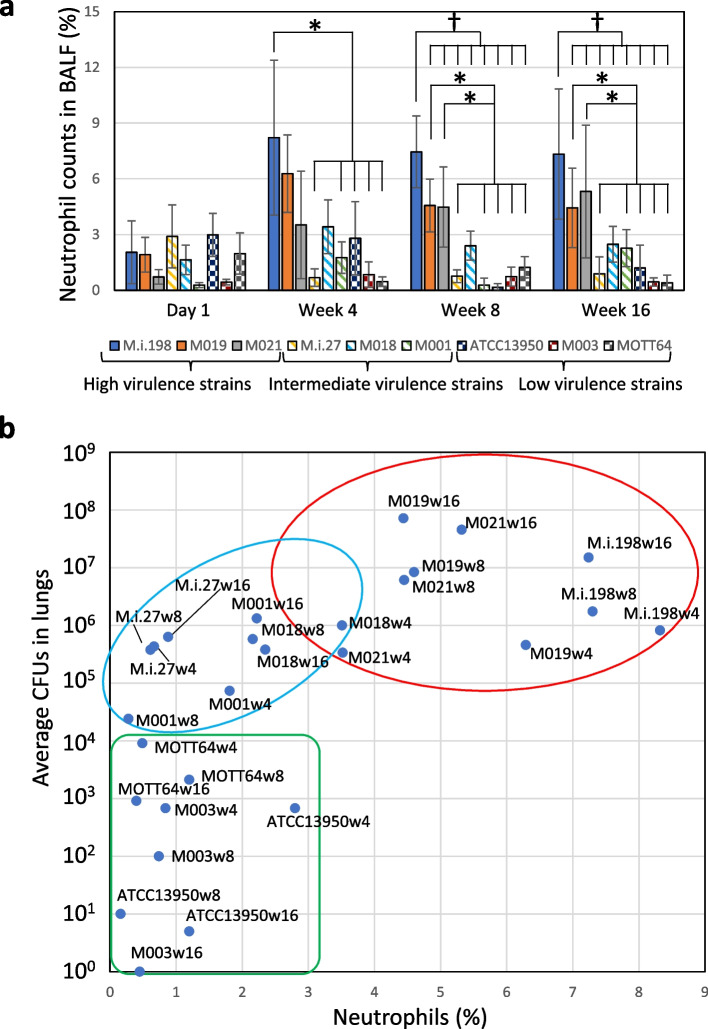
Relationship between neutrophilic infiltration and the bacterial load in the lungs of mice infected with *M. intracellulare* strains. **a** Comparison of the percentage of neutrophils in the BALF among strains. The bars are shaded by the virulence phenotypes as closed bars (high virulence strains), line-shaded bars (intermediate virulence strains) and dot-shaded bars (low virulence strains). *: Significant difference following infection with M.i.198 at 4 weeks of infection, and following infection with M019 and M021 at 8 weeks of infection, compared with M.i.27, M001, ATCC13950, M003, and MOTT64. †: Significant difference following infection with M.i.198 at 8 and 16 weeks of infection compared with any other strains. **b** Plotted data of the percentages for the average neutrophil counts and the CFUs in the lungs of mice infected with *M. intracellulare* strains. Red circle: high virulence strains, Blue circle: intermediate virulence strains, Green circle: low virulence strains. The numbers after the letter “w “ following strain name indicate sampling weeks, i.e. M003w16 indicates the samples of M003 at 16 weeks of infection

The bacterial load in the spleens infected with high virulence strains (M.i.198, M019 and M021) was increased along with the constant increase in the bacterial load in the lungs (Fig. 1). Whereas for the liver, following M021 infection the bacterial load increased significantly by 16 weeks, but following infection with other high virulence strains the bacterial load showed no significant difference. The high virulence strains induced peribronchovascular and alveolar inflammation histologically (Fig. 2, Additional files 4–8), and higher levels of neutrophilic infiltration in the bronchoalveolar lavage fluid (BALF) than the other strains (Fig. 3, Additional file 9). Neutrophilic infiltration in the BALF was higher following infection with M.i.198 than with two strains of intermediate virulence (M.i.27 and M001) and all low virulence strains after 4 weeks of infection, and M.i.198 induced the highest neutrophilic infiltration in the BALF among all other strains after 8 weeks of infection. Neutrophilic infiltration in the BALF was higher following infection with M019 and M021 than with the two strains of intermediate virulence (M.i.27 and M001) and the low virulence strains after 8 weeks of infection. In addition, the levels of lymphocytic infiltration in the BALF of the high virulence strains was higher, and reciprocally the proportion of monocytes/macrophages was lower following infection with the high virulence strains than with the low virulence strains after 8 weeks of infection (Additional files 9–10). In mice infected with these high virulence strains, neutrophilic lung inflammation was proposed to be related to the increased bacterial load (Fig. 3).

The bacterial load in the lungs infected with the two intermediate virulence strains (M.i.27 and M018) was maintained at a steady level during the 16 weeks of infection (Fig. 1). The bacterial load in the lungs infected with the other intermediate virulence strain M001 gradually decreased during the first 8 weeks of infection, then recovered by 16 weeks of infection to the same level as that at day 1 of infection. The bacterial loads in the spleens infected with M.i.27 and M018 were increased, but a similar increase was not observed following infection with M001. The bacterial loads in the livers infected with M018 were increased at 4 and 16 weeks compared with day 1 of infection, while the bacterial loads in the livers infected with other strains of intermediate virulence were not changed significantly during infection. Similar to the high virulence strains, M.i.27, M018, and M001 showed peribronchovascular and alveolar inflammation histologically (Fig. 2, Additional files 4–8). However, neutrophilic infiltration in the BALF was mild (1% to < 4% of the average percentage) in these intermediate virulence strains compared with the high virulence strains (Fig. 3). The proportion of infiltration with lymphocytes and macrophages varied between these intermediate virulence strains (Additional files 9–10).

The bacterial loads of the two low virulence strains (ATCC13950 and M003) showed a systemic decrease from 4 weeks of infection onwards, and were almost completely eliminated from 8 to 16 weeks of infection (Fig. 1). The bacterial load in the lungs infected with the other low virulence strain MOTT64 was gradually decreased during the 16 weeks of infection. The bacterial load in the spleens infected with MOTT64 increased by 4 weeks of infection, but showed no further increase after this time-point. The bacterial load in the livers infected with MOTT64 was almost unchanged during infection. Histological lung inflammation was evident at 4 weeks of infection with M003 and MOTT64, but was later resolved (Fig. 2, Additional files 4–8). The histology in the lungs infected with ATCC13950 was almost normal throughout infection. Except for mild neutrophilic infiltration in the BALF observed in mice infected with ATCC13950 at 4 weeks of infection, neutrophilic infiltration was negligible (less than 2% of the average percentage) following infection with all low virulence strains (Fig. 3b green circle).

### Survival assay

**Fig. 4:**
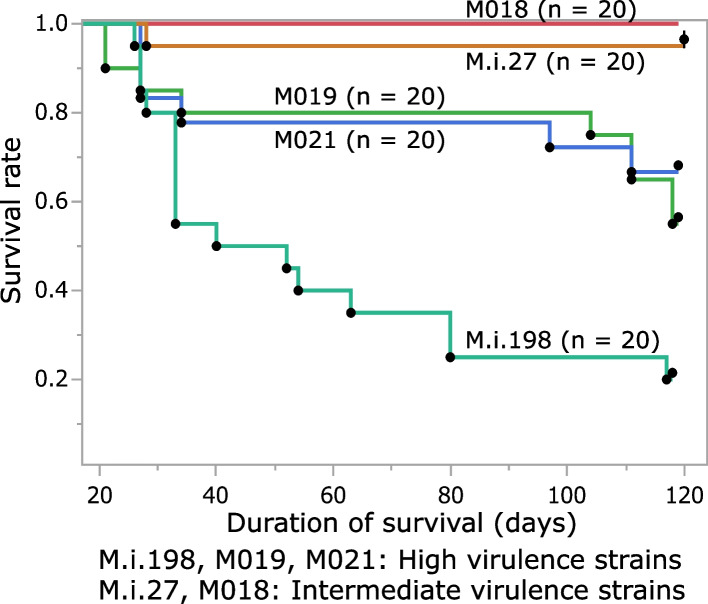
Kaplan–Meier survival analysis of mice infected with high-dose *M. intracellulare* strains (2 × 10^7^ CFUs). The prognosis in mice infected with M.i.198 was poorer than those infected with M.i.27 (*P* = 0.0011), M019 (*P* = 0.0195), and M021 (*P* = 0.0077). The prognosis in mice infected with M019 was poorer than those infected with M.i.27 (*P* = 0.0242). The prognosis in mice infected with M021 tended to be poorer than those infected with M.i.27 (*P* = 0.0548). All mice infected with M018 survived during infection

Although there was a difference in the kinetics of the bacterial load between strains, there was no loss of body weight in the infected mice (Additional file 11). However, prominent neutrophilic infiltration and sporadic cases of death occurred 4–6 weeks after mice were infected with the high virulence strain M.i.198, suggesting a certain impact of bacterial virulence on the prognosis of survival in the infected mice (Additional file 11). To clarify the relationship between virulence and mortality, we performed a survival assay in mice infected with a high inoculum (2 × 10^7^ CFUs) of bacterial strains, including three high virulence and two intermediate virulence strains. Mice infected with the high virulence strain M.i.198 displayed the most severe loss of body weight at 4 weeks of infection, and mortality dramatically increased after 4 weeks of infection, resulting in the worst prognosis among strains, which was a statistically significant result (Additional file 12, Fig. 4). Mice infected with the other two high virulence strains (M019, M021) showed a delayed gain in body weight compared with those infected with intermediate virulence strains (M018, M.i.27) (Additional file 12). The mortality rate among mice infected with M019 increased after 14 weeks of infection, resulting in a significantly poorer prognosis than those infected with M.i.27 (Fig. 4). The mice infected with M021 showed a similar prognostic curve to those infected with M019 and the prognosis tended to be poorer than those infected with M.i.27. No mortalities were recorded after 5 weeks of infection in mice infected with the intermediate virulence strains. These data confirmed the high virulence nature of M.i.198 and provided evidence of the lethal effects of chronic infection with high virulence *M. intracellulare* strains.

### Optimization of strains useful for chemotherapeutic experiments

Experimental infection with *M. intracellulare* strains causing bacterial replication and inflammation in the lungs is a promising model for testing the chemotherapeutic effects in vivo in MAC-PD. Clinical guidelines recommend CAM-containing multidrug therapy for MAC disease [4, 5]. However, there is little knowledge on the efficacy of each drug in eliminating bacteria and pathological improvements in vivo. In this study, the high virulence strain M019 was shown to be CAM-sensitive and induce a positive bacterial load with lung inflammation in mice. Therefore, mice infected with M019 may be applicable as a chemotherapeutic model in vivo.

**Fig. 5:**
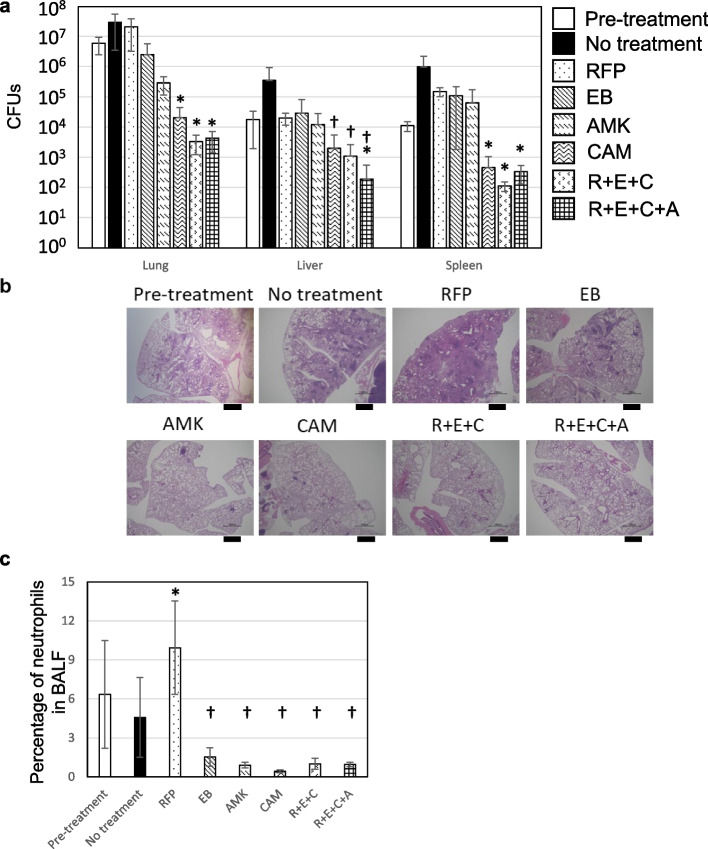
Effect of chemotherapy in M019-infected mice. **a** Comparison of the bacterial load before and after chemotherapy. At 4 weeks after infection, mice were divided into seven groups depending on the type of treatment. Data represent the mean ± SD of CFUs per organ. *: significantly different compared with AMK at 8 weeks after infection, †: significantly different compared with RFP at 8 weeks after infection. **b** Histological images of the lungs of M019-infected mice in each treatment group. Bars indicate 1 mm. (**c**) Comparison of neutrophilic infiltration in the BALF. *: significantly higher compared with before chemotherapy and the no-treatment group, †: significantly lower compared with before chemotherapy and the no-treatment group

Of the drugs tested in this study, CAM was the most effective at systemically decreasing the bacterial load (Fig. 5, Additional file 13). CAM induced a 2-log decrease in the bacterial load in the lungs, and a 1-log decrease in the bacterial load in the liver and spleen. CAM monotherapy induced scattered peribronchovascular inflammatory lesions, but the area and number of lesions were decreased compared with the no-treatment group. Combination therapy with rifampicin (RFP), ethambutol (EB), and CAM (R + E + C) induced a 3-log decrease in the bacterial load in the lungs, a 1-log decrease in the liver, and a 2-log decrease in the spleen compared with pre-treatment. Combination therapy with RFP, EB, CAM, and amikacin (R + E + C + A) induced a 3-log decrease in the bacterial load in the lungs, a 2-log decrease in the liver, and a 1.5-log decrease in the spleen. R + E + C + A decreased the bacterial load in the livers more effectively than any other regimen. Peribronchovascular inflammation was resolved following each of the multidrug regimens. Secondary to CAM, amikacin (AMK) was the next most effective drug for suppressing bacterial replication in the lungs. AMK monotherapy decreased the bacterial load by 1-log in the lungs and suppressed bacterial replication in the liver. Peribronchovascular inflammatory lesions remained after AMK monotherapy, but were milder than those in the no-treatment group. EB monotherapy effectively suppressed bacterial replication in the lungs and liver to pre-treatment levels. Scattered lesions of peribronchovascular inflammation were observed after EB monotherapy, similar to the no-treatment group. CAM-containing combination therapy and monotherapies of CAM, AMK, and EB significantly improved neutrophilic infiltration in the BALF (Fig. 5, Additional file 14).

RFP suppressed bacterial replication in the liver but not in the lungs or spleen (Fig. 5). While inflammation was evident in approximately half of the total lung area in the no-treatment group, RFP monotherapy resulted in inflammation of more than two-thirds of the total lung area (Fig. 5, Additional file 13). The immune cells infiltrating the lungs comprised predominantly of lymphocytes (56.8% of the average percentage), with an additional increase in neutrophils (9.93% of the average percentage). These chemotherapeutic data confirmed that M019-infected mice were an optimal model for evaluating therapeutic effects in vivo with respect to the high efficacy of CAM-containing combination regimens consistent with the clinical situation for MAC disease.

## Discussion

In contrast to *M. tuberculosis*, which shows homogeneous genetic and phenotypic characteristics [22], nontuberculous mycobacteria display high genetic and phenotypic diversity. A previous study examining the virulence of 41 MAC strains injected intravenously into mice revealed three virulence phenotypes, which was consistent with our current data [12]. Their samples were isolated from the environment as well as from infected humans and animals, while our samples were all clinical specimens from patients (ATCC13950 and MOTT64 were also isolated from patients and registered as type strains of *M. intracellulare* [TMI group] and *M. paraintracellulare* [MP-MIP group], respectively). Our data showed the obvious diversity of virulence of *M. intracellulare* even when the studied samples were limited to the human clinical strains. M.i.198 showed a consistent, highly virulent phenotype with respect to clinical disease progression and mortality in the mouse infection model, supporting the involvement of bacterial virulence in clinical disease progression [15]. However, for most of the *M. intracellulare* strains analyzed in this study, virulence in mice did not necessarily correspond to the genotype or clinical phenotype. Although antibiotic selection might occur in strains isolated from clinically progressive cases, a difference in growth in the lungs of infected mice was obvious between the high virulence strains and low virulence strains. In M003, for example, host factors may be expected to be involved in persistent infection in a human patient. Recently, a clinical study revealed a decrease in the estrogen level [23] and a genome-wide association study revealed a single nucleotide polymorphism pattern in the chromosomal region encoding the calcineurin-like DF-hand protein 2 (CHP2) [24]. These findings may provide insight into the mechanism of persistent infection. However, further studies are needed to identify the host factors involved to clarify how low virulence strains (lower than those of *M. tuberculosis*) are able to persist in the host. Evidence of the involvement of such host factors in disease susceptibility and progression may elucidate the molecular mechanism responsible for the onset of MAC-PD in the case of low virulence strains*.*

In tuberculosis, the most effective immunological response for containment of the tubercle bacilli is granuloma formation. In granuloma formation, Th1 cells play a key role in protective immunity by exerting IFN-γ signaling in mycobacterial diseases [7–10]. Recently, neutrophils have been suggested to play a role in enhancing the persistence of tubercle bacilli rather than exerting bactericidal activity [25–28]. In tuberculosis, neutrophils serve as a reservoir for the persistence of bacilli in human patients and neutrophilia corresponds to an increased risk of mortality, as revealed by a human population study [25, 29]. Furthermore, the accumulation of Ly6G-expressing granulocytes (mainly neutrophils) correlates with the bacterial load and disease severity in a mouse infection model [26]. In MAC-PD, the association between neutrophilic infiltration in the BALF and disease severity has been reported in clinical studies [27, 28]. In this study, mice infected with high virulence strains, especially the high virulence strain M.i.198, showed strong neutrophilic inflammation and high mortality, which supports the positive relationship between neutrophilic inflammation in the lungs and disease severity. The correlation between neutrophilic infiltration after 4 weeks of infection and the bacterial load in the lungs implicates the role of neutrophils as a niche environment allowing for the persistence of bacilli, similar to the case in tuberculosis (Fig. 3). The impact of neutrophilic infiltration on the containment of tubercle bacilli inside granulomas can be estimated by the computational granuloma simulator GranSim, which suggests that neutrophils facilitate local dissemination of granulomas and thereby the spread of infection mainly in the innate immune phase [30]. Although our model of intratracheal infection with 1 × 10^6^ CFUs of bacteria in C57BL/6 mice induced peribronchovascular inflammation composed of follicular lesions aggregated by mononuclear cells but no necrotic granulomatous lesions, neutrophilic infiltrations in the BALF were associated with phenotypic virulence in the infected mice. Thus, neutrophils may play a role in the insufficient containment of bacilli in granuloma lesions in advanced human MAC-PD.

The mechanism of neutrophilic infiltration in the chronic stages of infection has not been elucidated; however, it may be related to the tolerance of the Th1 immune response [31]. In tuberculosis, the Th1 immune response is decreased in the chronic stages of infection; thereby, tubercle bacilli escape the host immune response and persist in the host. Programmed cell death-1 (PD-1) and its ligand are key molecules of immunological tolerance in cancer [32]. However, the role of PD-1 is suggested to differ in tuberculosis. A recent study showed that PD-1 plays a protective role in the host by suppressing over-production of IFN-γ by Th1 cells [33]. Moreover, deletion of PD-1 and/or PD-L1 did not affect the growth of *M. avium* subspecies *hominissuis* in mice [34]. CD4^+^CD25^+^ regulatory T cells (Treg) are also considered to confer immunological tolerance because they express the transcription factor Foxp3, resulting in the suppression of IFN-γ and IL-2 production [35]. However, we found that the bacterial load and histopathology were similar regardless of the depletion of CD25^+^ cells at the later stage of infection in mice infected with *M. tuberculosis* [36]. Since the characteristics of immunological tolerance seem to differ between diseases, comprehensive studies including profiling of IFN-γ secretion from Th1 cells and neutrophilic infiltration at different stages of infection, under conditions of depletion of PD-1 and/or Treg, may provide insight into the different characteristics of immunological tolerance between MAC-PD and tuberculosis.

In this study, we developed a mouse model of chemotherapy by infection with the CAM-sensitive, high virulence strain M019. A previously reported mouse model of chemotherapy focused on *M. avium* 101 isolated from a patient with AIDS [37–40]. In that model, CAM decreased the bacterial load in the lungs (1.7 to 3.9-log) and spleen (0 to 0.84-log), which was consistent with our results. Furthermore, they showed that R + E + C decreased the bacterial load in the lungs (1.52 to 2.1-log), but to a lesser extent than in our model. The smaller improvement in the bacterial load in mice infected with *M. avium* 104 than with *M. intracellulare* M019 may be related to the tendency for higher antibiotic susceptibility in *M. intracellulare* than in *M. avium* [16] and the difference of the ability to persist preferentially in different host organs between *M. avium* and *M. intracellulare*.

In our study, with the exception of RFP, monotherapies were shown to effectively suppress bacterial growth in the lungs. After CAM, AMK injection was the next most effective drug at reducing the bacterial load in the lungs, and AMK showed additional bactericidal effects in the liver when used as a combination therapy. By contrast, RFP monotherapy exacerbated lung inflammation histologically (Fig. 5, Additional file 13), at the same bacterial load as the no-treatment group. This was consistent with the initial aggravation of symptoms sometimes observed at the early stage of chemotherapy in cases of tuberculosis [41]. During this initial aggravation, inflammation is exacerbated, which does not match the normal course of improvement during anti-tuberculous chemotherapy containing isoniazid and RFP [41]. This phenomenon has long been understood clinically as a local allergic reaction to dead bacteria and an immune response imbalance. However, more recently, it has been regarded as immunologically similar to immune reconstitution inflammatory syndrome (IRIS), which can be induced by restarting highly active anti-retroviral therapy against human immunodeficiency virus (HIV) infection and the discontinuation of tumor necrosis factor-alpha (TNF-α) inhibitor therapy against rheumatoid arthritis and other autoimmune diseases [42, 43]. Although the detailed mechanism of exacerbation of inflammation induced by RFP monotherapy was not proven in this study, one proposed mechanism is the increased activation of CD8^+^ cytotoxic T cells, as suggested by a clinical study of predictors of *M. avium-intracellulare* complex IRIS in patients with HIV [44]. Since combination therapy did not induce exacerbation of inflammation, combination therapy is considered to prevent such an excessive immune reaction, whilst also effectively eliminating bacteria in clinical MAC-PD.

Although the overall structure of the immune system in mice and humans is similar, some differences of immunology have been revealed between mice and humans [45]. The difference of immunology between species of the hosts may explain the deviation of infection outcome in mice and clinical outcome in humans for the same strains observed in this study. First, as for the balance of lymphocytes and neutrophils, mouse blood has a predominance of lymphocytes, while human blood is rich in neutrophils [46]. Bronchus-associated lymphoid tissue is found in mice but this is largely absent in healthy humans [47]. There are differences in the chemokines and their receptors between humans and mice. For example, the important molecules for neutrophil chemotaxis IL-8 (CXC motif chemokine ligand 8; CXCL8) and its receptor CXC motif chemokine receptor 1 (CXCR1) are present in humans but not in mice. Second, the mechanism of antimycobacterial activity is different between mice and humans. In mouse macrophages, nitric oxide is synthesized by stimulation with IFN-γ, which plays a major role in antimycobacterial activity [48]. While, in human macrophages, Toll-like receptor signaling plays a major role in the production of antimicrobial peptides [49, 50]. Third, Th1/Th2 polarization is relatively clear in mice but the paradigm has not been clear-cut in humans [45]. For example, the inhibitory cytokine IL-10 is produced only from Th2 cells in mice, while IL-10 is produced both from Th1 and Th2 cells in humans. Further studies are needed to clarify the immunological pathogenesis of MAC-PD by focusing on the human-specific immunological system, for example by using humanized mice for immunology.

There are some limitations to this study. First, this study identified several virulent strains of *M. intracellulare* but did not identify the specific virulence factors involved in disease progression. Although the development of gene manipulation technology has been delayed, we have established a transposon mutagenesis system [51] with which we are constructing deletion mutants of *M. intracellulare*. Furthermore, the genomes of the clinical strains used in this study have been completely sequenced [19]. As a next step, we plan to identify the virulence factors for disease progression by infecting mice with the genetically-manipulated strains. Second, the number of strains was insufficient to elucidate the complete relationship between genotypes and virulence phenotypes. However, increasing the number of strains would make such a study laborious, particularly if individual mice were used for each time-point, similar to this study. Recently, an in vivo live imaging system has been reported to monitor infection dynamics in individual animals infected with luciferase-expressing microbes [52]. This imaging system may facilitate comparisons of the virulence of an increasing number of strains of different genotypes.

In conclusion, the virulence of clinical *M. intracellulare* strains in mice was able to be classified into three groups: high, intermediate, and low. Neutrophilic inflammation in the lungs was associated with disease severity in mice. In particular, the hypervirulence of M.i.198 supported the involvement of bacterial virulence in disease progression. Furthermore, we established a mouse infection model for evaluating chemotherapeutic efficacy in vivo using a high virulence strain. These data provide basic information on the virulence of clinical *M. intracellulare* strains and a strategy for the evaluation of novel drug efficacy.

## Materials and methods

### Bacterial strains

Seven clinical strains isolated from non-AIDS patients with MAC-PD and two type strains of *M. intracellulare* ATCC13950 (belonging to TMI group) and *M. paraintracellulare* MOTT64 (belonging to MP-MIP group) were used in this study (Table 1). All clinical strains were isolated at NHO Toneyama Medical Center. Informed consent was obtained from all patients according to the guidelines of the Institutional Review Board of NHO Toneyama Medical Center and Niigata University. The clinical strains included five strains used for the previous comparative genome analysis (M001, M003, M018, M019, and M021) [19] and two strains used for the previous infection experiment in mice (M.i.198 and M.i.27) [15]. Diagnosis of MAC-PD was made according to the American Thoracic Society guidelines [4, 5].

### Animals

Female 6-week-old C57BL/6JJcl mice were purchased from CLEA Japan (Tokyo, Japan). All mice were kept under specific-pathogen-free conditions in the animal facility of Niigata University Graduate School of Medicine according to the institutional guidelines for animal experiments. Mice were housed (4 per cage) under a 12-h/12-h light/dark cycle (lights on between 08:00 and 20:00). The intake of food and water was ad libitum. Euthanasia was conducted by the intraperitoneal injection of a combination anesthetic (15 μg of medetomidine, 80 μg of midazolam, and 100 μg of butorphanol) followed by cervical dislocation.

### Drugs

RFP was purchased from FUJIFILM Wako Pure Chemical (Osaka, Japan). EB, CAM and AMK were purchased from Tokyo Kasei (Tokyo, Japan). For the chemotherapeutic experiment, these drugs were dissolved in sterile water containing 2.5% gum Arabic and 0.05% Tween 80. The solutions were stored at 4 °C for up to 1 week.

### MIC assay

The MIC of clarithromycin was measured by inoculating the bacteria in Middlebrook 7H9 medium (Difco Laboratories, Detroit, MI) containing 0.2% glycerol, 0.1% Tween 80 (MP Biomedicals, Illkirch, France) and 10% Middlebrook albumin-dextrose-catalase (7H9/ADC/Tween 80) (OD_600_ 0.003) in 96-well plates followed by incubation at 37 °C for 2 weeks.

### Infection experiment

Twenty-four mice were used per strain for the assay. The amounts of bacteria contained in bacterial suspension used for infection experiment were justified by CFU count before assay. Fifty microliters of bacterial suspension containing 1 × 10^6^ CFUs of bacteria were inoculated intratracheally under the combination anesthesia (medetomidine 0.75 mg kg^−1^, midazolam 4 mg kg^−1^, butorphanol 5 mg kg^−1^ intraperitoneally). On day 1, and 4, 8, 16 weeks after inoculation, mice were sacrificed by cervical dislocation under the combination anesthesia, and lungs, spleen, and liver were harvested. For five mice per strain, the organs were homogenized in 4.5 mL of distilled water by a gentleMACS™ dissociator (Miltenyi Biotec, Bergisch Gladbach, Germany) at each time-point, and 0.2 mL of tenfold dilutions of the homogenates were plated on 7H10-OADC agar followed by cultivation for 3 weeks. The bacterial load was evaluated by determining the CFUs per organ. For one mouse per strain, histological sections were prepared at each time-point by standard methods, including formalin fixation, dehydration, embedding in paraffin, and staining with hematoxylin and eosin.

### Bronchoalveolar lavage (BAL)

Before removing the organs, all mice underwent BAL for total lung lavage. BAL was performed by instilling five 1-mL aliquots of sterilized normal saline into the trachea through a 24G indwelling tube under the combination anesthesia. Then 1.5 mL of the obtained fluid was centrifuged at 800 × *g* for 5 min at 4 °C, the cell pellets were dissolved in 0.5 mL of normal saline and cells were counted using a hemocytometer. The suspension was centrifuged by a cytospin. After staining of cell pellets with May–Grünwald Giemsa, differential cell counts were performed by examining 500 cells using a standard light microscope (Nikon ECLIPSE Ci-L, Tokyo, Japan). Histopathological images were captured by a KEYENCE BZX microscope (KEYENCE BZ-X700, Osaka, Japan).

### Survival experiment

Twenty mice were used per strain for the survival assay. Fifty microliters of bacterial suspension containing 2 × 10^7^ CFUs of bacteria were inoculated intratracheally under the combination anesthesia. Two mice infected with M021 were excluded from the analysis because they died within 24 h of infection (Additional file 12). This was thought to be because of a problem with inoculation rather than a reflection of virulence. All animals were monitored biweekly, for at least 5 days per week, for clinical signs of disease. Animals were euthanized if they exhibited > 25% weight loss in 7 days, as approved by the institutional guidelines for animal experiments.

### Chemotherapy

Eight mice were used per group and were exposed to therapeutic regimens. Fifty microliters of bacterial suspension containing 1 × 10^6^ CFUs of M019 were inoculated intratracheally. At 4 weeks of infection, administration of drugs was started. Two hundred microliters of RFP at 10 mg kg^−1^, EB at 100 mg kg^−1^, and CAM at 100 mg kg^−1^ was instilled through an esophageal cannula, and AMK at 100 mg kg^−1^ was injected subcutaneously six times per week. Two hundred microliters of the vehicle were instilled in the non-treatment mice. Following a previous report, RFP was administered 1 h prior to the administration of the other drugs to avoid pharmacokinetic interactions [40]. After 4 weeks of chemotherapy (8 weeks of infection), the lungs, livers, and spleens were harvested to evaluate the bacterial load in 5–6 mice and for histological examination in 1–2 mice. To avoid the carryover effects of the drugs in the organs, treated mice were euthanized 48 to 144 h after administration of the last dose of treatment.

### Statistical analysis

Statistical analysis was performed using JMP software (SAS Institute Inc., NC, USA). The data of CFUs after infection for each strain were compared between time-points using a paired *t*-test. Comparison of the data of CFUs after chemotherapy between treatment groups and the percentage of differential counts for the BALF between strains were performed between time-points using ANOVA with Tukey’s HSD test. The duration of survival was calculated by Kaplan–Meier analysis, and statistical significance was determined with the log rank (Mantel–Cox) test. The data were shown as the means ± standard deviations. A significant difference was set as *P* < 0.05.

## Supplementary Information

**Additional file 1: Fig. S1.** Phylogenetic position of the strains used in this study. The strains used in this study are designated by the red square. The clinical strains isolated from *M. intracellulare* pulmonary disease are classified into two groups: (1) typical *M. intracellulare* group (TMI), (2) *M. paraintracellulare* (MP)-*M. indicus pranii* (MIP) group [Reference 19].

**Additional file 2: Table S1.** Clinical features of *M. intracellulare* strains used in this study.

**Additional file 3.** Description of the samples showing no colonies recovered in low virulence strains.

**Additional file 4: Fig S2.** Histological images of the lungs during 16 weeks of infection in C57BL/6 mice by hematoxylin-eosin staining. Bars indicate 100 μm.

**Additional file 5: Fig. S3.** Histological images of the lungs at day 1 of infection in C57BL/6 mice by hematoxylin-eosin staining. Bars indicate 50 μm.

**Additional file 6: Fig. S4.** Histological images of the lungs at 4 weeks of infection in C57BL/6 mice by hematoxylin-eosin staining. Bars indicate 50 μm.

**Additional file 7: Fig. S5.** Histological images of the lungs at 8 weeks of infection in C57BL/6 mice by hematoxylin-eosin staining. Bars indicate 50 μm.

**Additional file 8: Fig. S6.** Histological images of the lungs at 16 weeks of infection in C57BL/6 mice by hematoxylin-eosin staining. Bars indicate 50 μm.

**Additional file 9: Table S2.** Bronchoalveolar lavage fluid (BALF) counts in mice infected with *M. intracellulare* strains.

**Additional file 10: Fig. S7.** Relationship between the infiltration of monocytes/macrophages and lymphocytes in the BALF and the bacterial load in the lungs infected with *M. intracellulare* strains. **a** Comparison of the percentage of monocytes/macrophages (right) and lymphocytes (left) in the BALF among infecting strains. The proportions of monocytes/macrophages and lymphocytes were lower and higher, respectively, in the BALF with high virulence strains (M.i.198, M019, M021) than with low virulence strains (ATCC13950, M003, MOTT64) after 8 weeks of infection. **b** Plotted data of the percentage of average macrophage and lymphocyte counts and the CFUs in lungs infected with *M. intracellulare* strains.

**Additional file 11: Fig. S8.** Data of the changes of body weight and survival rate following infection with 1×10^6^ CFUs of *M. intracellulare* strains. **a** Time-course of the changes in body weight in mice infected with *M. intracellulare* strains. **b** Survival curve of mice infected with M.i.198. Mortality sporadically occurred during 4–6 weeks of infection.

**Additional file 12: Fig. S9.** Data of the changes of body weight following infection with 2×10^7^ CFUs of *M. intracellulare* strains. **a** Time-course of the changes in body weight in mice infected with *M. intracellulare* strains. There was a significant difference in body weight in M.i.198-infected mice at 4 weeks of infection compared with mice infected with M.i.27, M018, M019 and M021 and the no-infection group. The body weight of mice infected with M019 was lower than that of mice infected with the intermediate virulence strains (M.i.27, M018) after 4 weeks of infection. The body weight of mice infected with M019 was lower than that of mice infected with M021 at 16 weeks of infection. The body weight of mice infected with M021 was lower than that of mice infected with the intermediate strains after 8 weeks of infection. Data on body weight was limited to surviving mice at the time-points of measurement. **b** The number of mice assayed for the measurement of body weight at each time-point. * Twenty mice were prepared at day 0 of the experiment, but two mice died the day after intratracheal instillation.

**Additional file 13: Fig. S10.** BALF and histological findings on the chemotherapeutic experiment. **a** Comparison of the infiltration of macrophages/monocytes (Right) and lymphocytes (Left) in the BALF before and after chemotherapy. *: significantly lower compared with all other groups and before chemotherapy, #: significantly higher compared with CAM monotherapy, RFP+EB+CAM (R+E+C), RFP+EB+CAM+AMK (R+E+C+A), and before chemotherapy. †: significantly higher compared with CAM monotherapy, R+E+C, and R+E+C+A. ‡: significantly higher compared with R+E+C+A. **b** Histological images of the lungs of M019-infected mice for each treatment group. Bars indicate 100 μm.

**Additional file 14: Table S3.** Bronchoalveolar lavage fluid (BALF) counts in mice infected with M019 and treated with chemotherapy.

## Data Availability

The datasets used/or analyzed during the current study are available from the corresponding author on reasonable request.
