# Supplementary material for: Virulence of Mycobacterium intracellulare clinical strains in a mouse model of lung infection – role of neutrophilic inflammation in disease severity
Source: BMC Microbiol. 2023 Apr 3;23:94. doi: 10.1186/s12866-023-02831-y (PMC10069106; doi:10.1186/s12866-023-02831-y)
Supplement: Supplementary file 1 — Additional file 1: Fig. S1. Phylogenetic position of the strains used in this study. The strains used in this study are designated by the red square. The clinical strains isolated from M. intracellulare pulmonary disease are classified into two groups: (1) typical M. intracellulare group (TMI), (2) M. paraintracellulare (MP)-M. indicus pranii (MIP) group [Reference 19]. [file 12866_2023_2831_MOESM1_ESM.pptx]

## Slide 1
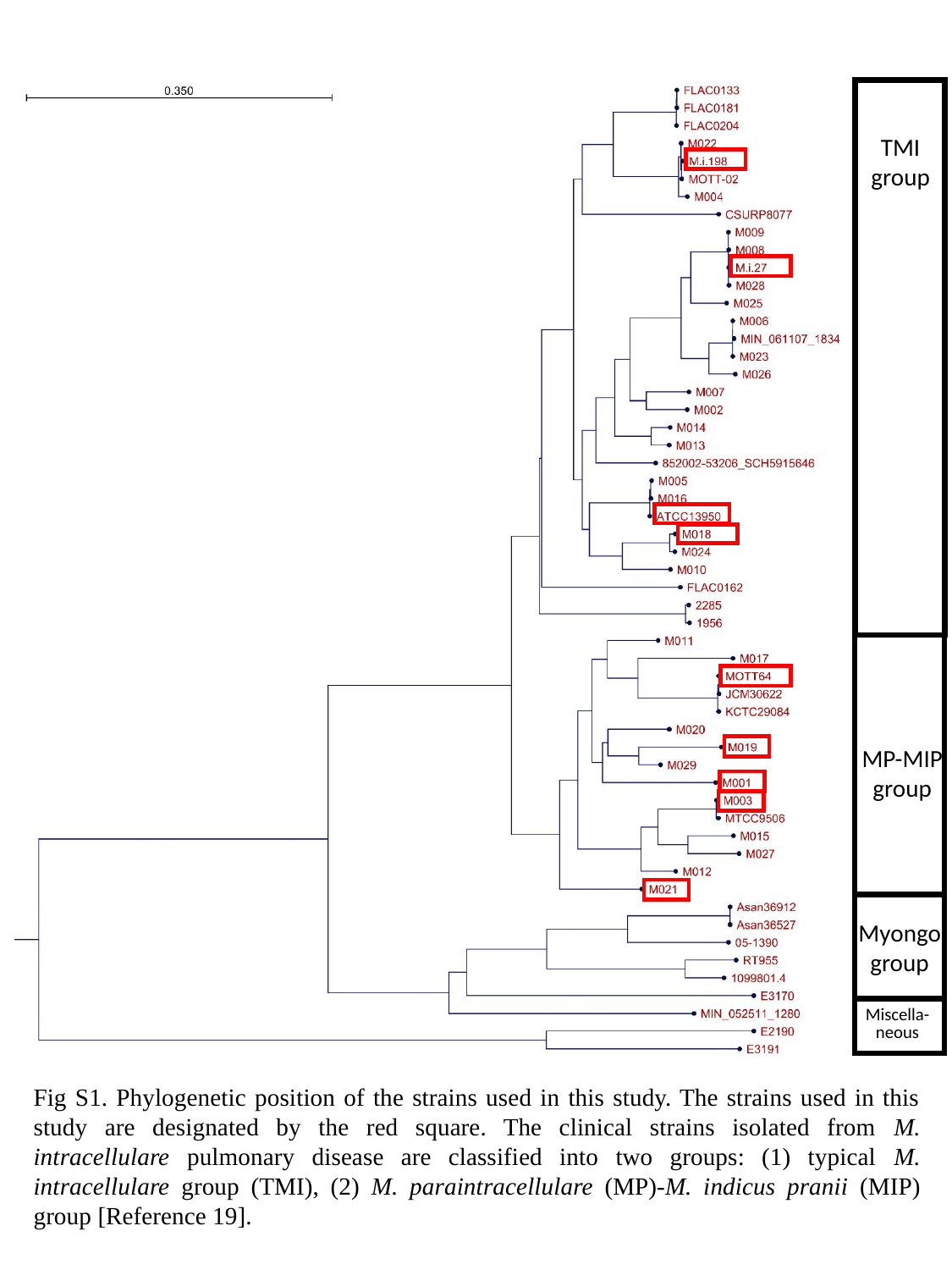

TMI group
MP-MIP group
Myongo group
Miscella-neous
Fig S1. Phylogenetic position of the strains used in this study. The strains used in this study are designated by the red square. The clinical strains isolated from M. intracellulare pulmonary disease are classified into two groups: (1) typical M. intracellulare group (TMI), (2) M. paraintracellulare (MP)-M. indicus pranii (MIP) group [Reference 19].
