# Supplementary material for: Virulence of Mycobacterium intracellulare clinical strains in a mouse model of lung infection – role of neutrophilic inflammation in disease severity
Source: BMC Microbiol. 2023 Apr 3;23:94. doi: 10.1186/s12866-023-02831-y (PMC10069106; doi:10.1186/s12866-023-02831-y)
Supplement: Supplementary file 2 — Additional file 2: Table S1. Clinical features of M. intracellulare strains used in this study. [file 12866_2023_2831_MOESM2_ESM.docx]

**Table S1.** Clinical features of *M. intracellulare* strains used in this study.

| Strain | Genotype^a^ | Clinical phenotype | Age | Sex | Erythrocyte sedimentation rate (mm/h) | Chest X-ray findings | | | Sputum |  | Duration of years from onset to sputum sampling | MIC value of clarithromycin (μg/mL) | References |
| --- | --- | --- | --- | --- | --- | --- | --- | --- | --- | --- | --- | --- | --- |
|  |  |  |  |  |  | Type^b^ | Extent^c^ | Progression of radiographic findings^c^ | Smear | Culture |  |  |  |
| ATCC13950 | TMI | Type strain of TMI genotype |  |  |  |  |  |  |  |  |  |  |  |
| M.i.198 | TMI | Progressive | 62 | F | 108 | FC | Far advanced | Progressive | 2+ | 2+ | 3 | 160< | 15 |
| M.i.27 | TMI | Stable | 67 | F | 50 | NC-NB | Moderate | Stable | (-) | 1+ | 17 | 0.156> | 15 |
| M018 | TMI | Stable | 67 | F | 41 | C-NB | Moderate | Slightly progressive | (-) | 1+ | 12 | 0.156> | 19 |
| MOTT64 | MP-MIP | Type strain of MP-MIP genotype |  |  |  |  |  |  |  |  |  |  |  |
| M001 | MP-MIP | Progressive | 90 | M | 58 | C-NB | Far advanced | Progressive | 2+ | 2+ | 18 | 160< | 19 |
| M003 | MP-MIP | Progressive | 72 | F | 77 | C-NB | Far advanced | Progressive | 2+ | 2+ | 21 | 20 | 19 |
| M019 | MP-MIP | Stable | 83 | M | 20 | NC-NB | Moderate | Stable | (-) | 1+ | 10 | 0.156> | 19 |
| M021 | MP-MIP | Stable | 68 | M | 55 | C-NB | Minimal | Stable | (-) | 1+ | 3 | 0.156> | 19 |

^a^TMI; typical *Mycobacterium intracellulare* genotype; MP-MIP: *Mycobacterium paraintracellulare-M. indicus pranii* genotype.

^b^FC: fibrocavitary disease; NC-NB: noncavitary nodular bronchiectatic disease; C-NB: cavitary nodular bronchiectatic disease.

^c^Extent and progression of radiographic findings was graded according to our previous report^20^; minimal: total area of abnormal shadows within the one-third of the unilateral lung area, moderate: total area of abnormal shadows beyond the one-third and within the unilateral lung; far advanced: total area of abnormal shadows beyond the unilateral lung area; stable, shrinkage or unchanged of abnormal shadows; slightly progressive, a slight increase in the size of preexisting abnormal shadows; progressive, emergence of consolidation or cavitary lesions in addition to an increase in the size of preexisting abnormal shadows.
