# Supplementary material for: Virulence of Mycobacterium intracellulare clinical strains in a mouse model of lung infection – role of neutrophilic inflammation in disease severity
Source: BMC Microbiol. 2023 Apr 3;23:94. doi: 10.1186/s12866-023-02831-y (PMC10069106; doi:10.1186/s12866-023-02831-y)
Supplement: Supplementary file 3 — Additional file 3. Description of the samples showing no colonies recovered in low virulence strains. [file 12866_2023_2831_MOESM3_ESM.docx]

Additional file 3: Description of the samples showing no colonies recovered in low virulence strains.

Of 5 mice sacrificed for CFU assay in ATCC13950, no colonies were recovered from the liver and spleen of 1 mouse at 1-day of infection. No colonies were recovered from the lungs and liver of 1 mouse, from the spleen of 4 mice at 4 weeks of infection. No colonies were recovered from the lungs of 4 mice, from the liver of 5 mice, from the spleen of 5 mice at 8 and 16 weeks of infection.

Of 5 mice sacrificed for CFU assay in M003, no colonies were recovered from the spleen of 1 mouse at 1-day of infection. No colonies were recovered from the lungs and liver of 1 mouse, from the spleen of 4 mice at 4 weeks of infection. No colonies were recovered from the lungs of 4 mice, from the liver of 3 mice, from the spleen of 4 mice at 8 weeks of infection. No colonies were recovered from the lungs of 5 mice, from the liver of 5 mice, from the spleen of 5 mice at 16 weeks of infection.

Of 5 mice sacrificed for CFU assay in MOTT64, no colonies were recovered from the liver of 1 mouse, from the spleen of 4 mice at 1-day of infection. No colonies were recovered from the liver of 1 mouse. No colonies were recovered from the lungs, liver and spleen of 1 mouse at 8 weeks of infection. No colonies were recovered from the lungs of 2 mice, from the spleen of 3 mice at 16 weeks of infection.
