# Supplementary material for: Virulence of Mycobacterium intracellulare clinical strains in a mouse model of lung infection – role of neutrophilic inflammation in disease severity
Source: BMC Microbiol. 2023 Apr 3;23:94. doi: 10.1186/s12866-023-02831-y (PMC10069106; doi:10.1186/s12866-023-02831-y)
Supplement: Supplementary file 4 — Additional file 4: Fig S2. Histological images of the lungs during 16 weeks of infection in C57BL/6 mice by hematoxylin-eosin staining. Bars indicate 100 μm. [file 12866_2023_2831_MOESM4_ESM.pptx]

## Slide 1
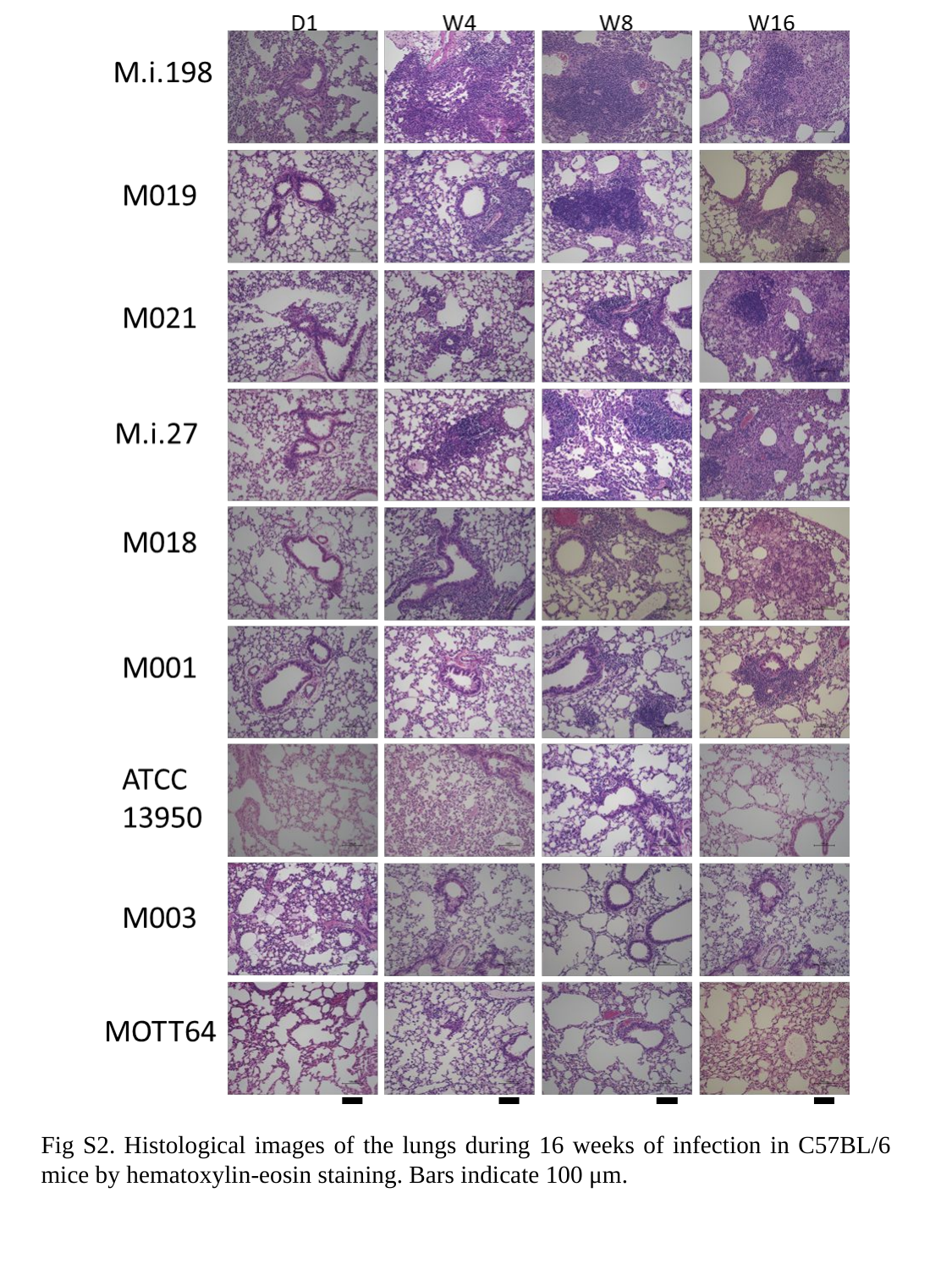

Fig S2. Histological images of the lungs during 16 weeks of infection in C57BL/6 mice by hematoxylin-eosin staining. Bars indicate 100 μm.
