# Supplementary material for: Virulence of Mycobacterium intracellulare clinical strains in a mouse model of lung infection – role of neutrophilic inflammation in disease severity
Source: BMC Microbiol. 2023 Apr 3;23:94. doi: 10.1186/s12866-023-02831-y (PMC10069106; doi:10.1186/s12866-023-02831-y)
Supplement: Supplementary file 6 — Additional file 6: Fig. S4. Histological images of the lungs at 4 weeks of infection in C57BL/6 mice by hematoxylin-eosin staining. Bars indicate 50 μm. [file 12866_2023_2831_MOESM6_ESM.pptx]

## Slide 1
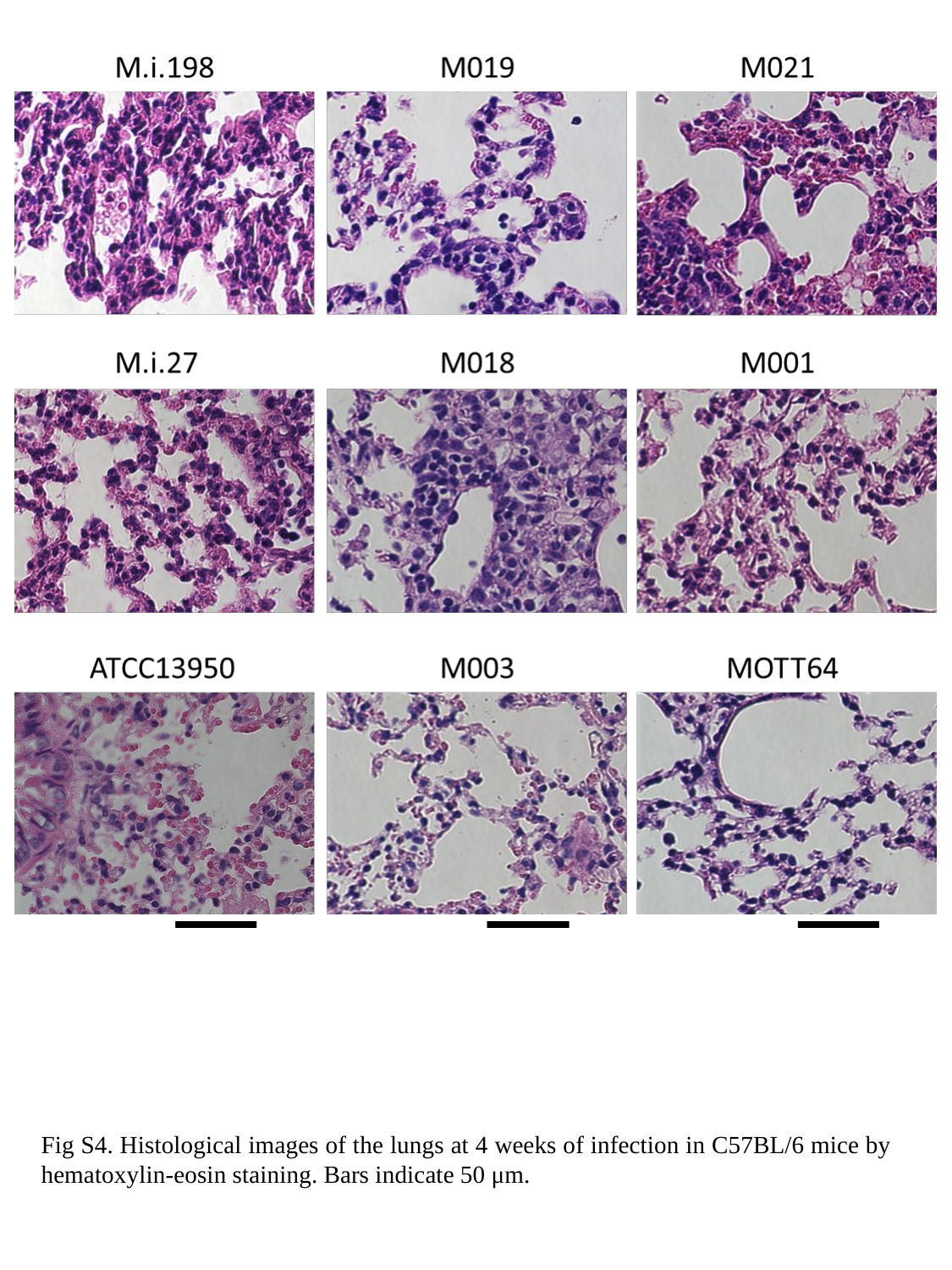

Fig S4. Histological images of the lungs at 4 weeks of infection in C57BL/6 mice by hematoxylin-eosin staining. Bars indicate 50 μm.
