# Supplementary material for: Virulence of Mycobacterium intracellulare clinical strains in a mouse model of lung infection – role of neutrophilic inflammation in disease severity
Source: BMC Microbiol. 2023 Apr 3;23:94. doi: 10.1186/s12866-023-02831-y (PMC10069106; doi:10.1186/s12866-023-02831-y)
Supplement: Supplementary file 10 — Additional file 10: Fig. S7. Relationship between the infiltration of monocytes/macrophages and lymphocytes in the BALF and the bacterial load in the lungs infected with M. intracellulare strains. a Comparison of the percentage of monocytes/macrophages (right) and lymphocytes (left) in the BALF among infecting strains. The proportions of monocytes/macrophages and lymphocytes were lower and higher, respectively, in the BALF with high virulence strains (M.i.198, M019, M021) than with low virulence strains (ATCC13950, M003, MOTT64) after 8 weeks of infection. b Plotted data of the percentage of average macrophage and lymphocyte counts and the CFUs in lungs infected with M. intracellulare strains. [file 12866_2023_2831_MOESM10_ESM.pptx]

## Slide 1
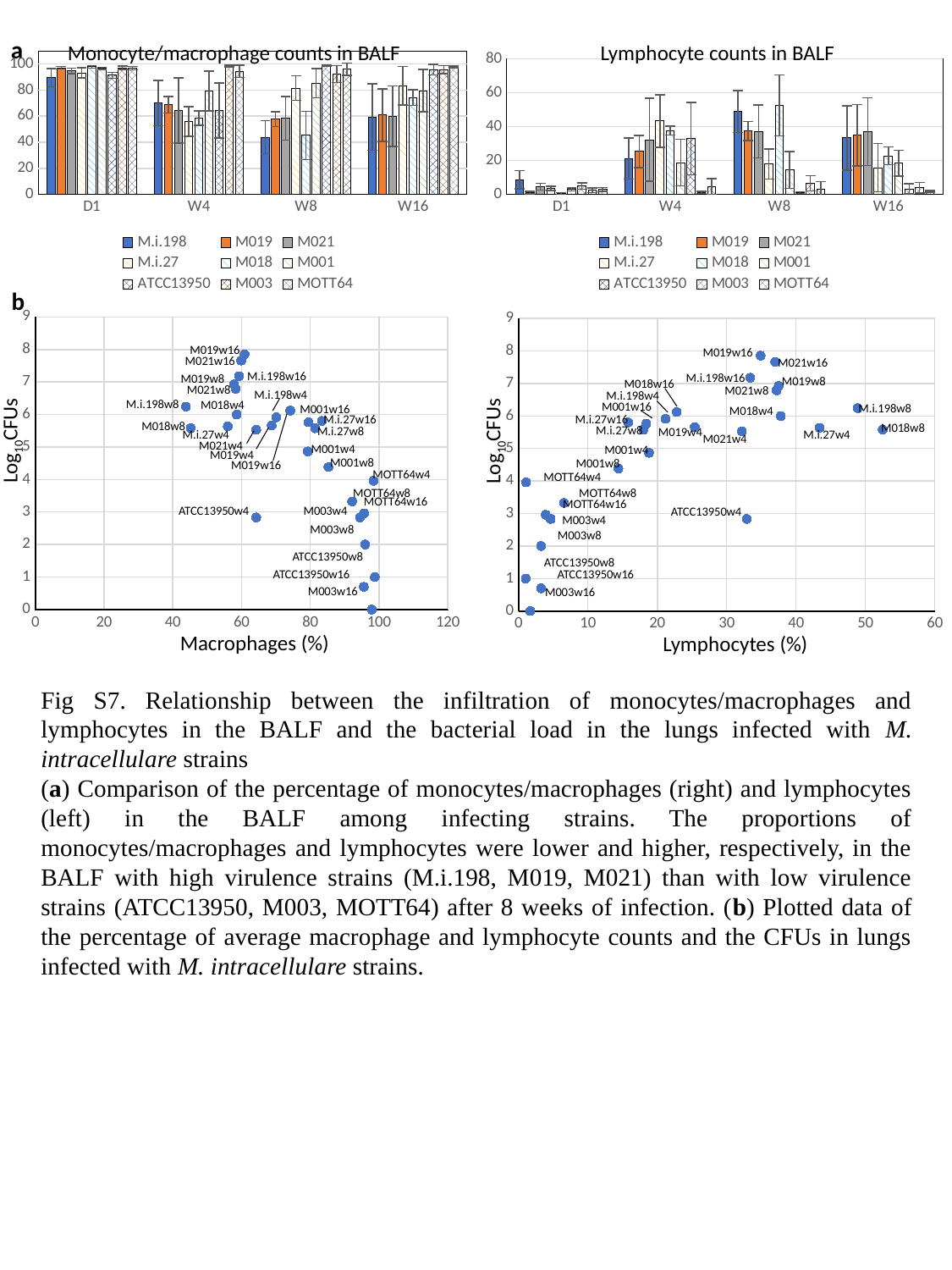

a
Monocyte/macrophage counts in BALF
Lymphocyte counts in BALF
### Chart
| Category | M.i.198 | M019 | M021 | M.i.27 | M018 | M001 | ATCC13950 | M003 | MOTT64 |
|---|---|---|---|---|---|---|---|---|---|
| D1 | 89.4 | 97.0 | 94.7 | 93.2 | 97.8 | 96.6 | 91.3 | 97.1 | 96.7 |
| W4 | 70.1 | 68.8 | 64.3 | 56.0 | 58.6 | 79.3 | 64.3 | 98.4 | 94.5 |
| W8 | 43.8 | 57.8 | 58.3 | 81.4 | 45.2 | 85.3 | 98.8 | 92.2 | 96.0 |
| W16 | 59.3 | 60.9 | 59.9 | 83.4 | 74.2 | 79.5 | 95.6 | 95.7 | 97.9 |
### Chart
| Category | M.i.198 | M019 | M021 | M.i.27 | M018 | M001 | ATCC13950 | M003 | MOTT64 |
|---|---|---|---|---|---|---|---|---|---|
| D1 | 8.6 | 1.0 | 4.56 | 3.6 | 0.56 | 3.0 | 4.83 | 2.48 | 2.8 |
| W4 | 21.2 | 25.4 | 32.2 | 43.4 | 37.8 | 18.8 | 32.9 | 1.09 | 4.64 |
| W8 | 48.9 | 37.5 | 37.2 | 18.0 | 52.5 | 14.4 | 1.04 | 6.56 | 3.24 |
| W16 | 33.4 | 34.9 | 37.0 | 15.8 | 22.8 | 18.4 | 3.24 | 3.92 | 1.7 |b
### Chart
| Category | CFU |
|---|---|
### Chart
| Category | CFU |
|---|---|M019w16
M019w16
M021w16
M021w16
M.i.198w16
M.i.198w16
M019w8
M019w8
M018w16
M021w8
M021w8
M.i.198w4
M.i.198w4
M.i.198w8
M018w4
M001w16
M.i.198w8
M001w16
M018w4
M.i.27w16
M.i.27w16
M018w8
M018w8
M.i.27w8
M.i.27w8
M019w4
M.i.27w4
M.i.27w4
Log10CFUs
Log10CFUs
M021w4
M021w4
M001w4
M001w4
M019w4
M001w8
M001w8
M019w16
MOTT64w4
MOTT64w4
MOTT64w8
MOTT64w8
MOTT64w16
MOTT64w16
M003w4
ATCC13950w4
ATCC13950w4
M003w4
M003w8
M003w8
ATCC13950w8
ATCC13950w8
ATCC13950w16
ATCC13950w16
M003w16
M003w16
Macrophages (%)
Lymphocytes (%)
Fig S7. Relationship between the infiltration of monocytes/macrophages and lymphocytes in the BALF and the bacterial load in the lungs infected with M. intracellulare strains
(a) Comparison of the percentage of monocytes/macrophages (right) and lymphocytes (left) in the BALF among infecting strains. The proportions of monocytes/macrophages and lymphocytes were lower and higher, respectively, in the BALF with high virulence strains (M.i.198, M019, M021) than with low virulence strains (ATCC13950, M003, MOTT64) after 8 weeks of infection. (b) Plotted data of the percentage of average macrophage and lymphocyte counts and the CFUs in lungs infected with M. intracellulare strains.
