# Supplementary material for: Virulence of Mycobacterium intracellulare clinical strains in a mouse model of lung infection – role of neutrophilic inflammation in disease severity
Source: BMC Microbiol. 2023 Apr 3;23:94. doi: 10.1186/s12866-023-02831-y (PMC10069106; doi:10.1186/s12866-023-02831-y)
Supplement: Supplementary file 11 — Additional file 11: Fig. S8. Data of the changes of body weight and survival rate following infection with 1×10^6^ CFUs of M. intracellulare strains. a Time-course of the changes in body weight in mice infected with M. intracellulare strains. b Survival curve of mice infected with M.i.198. Mortality sporadically occurred during 4–6 weeks of infection. [file 12866_2023_2831_MOESM11_ESM.pptx]

## Slide 1
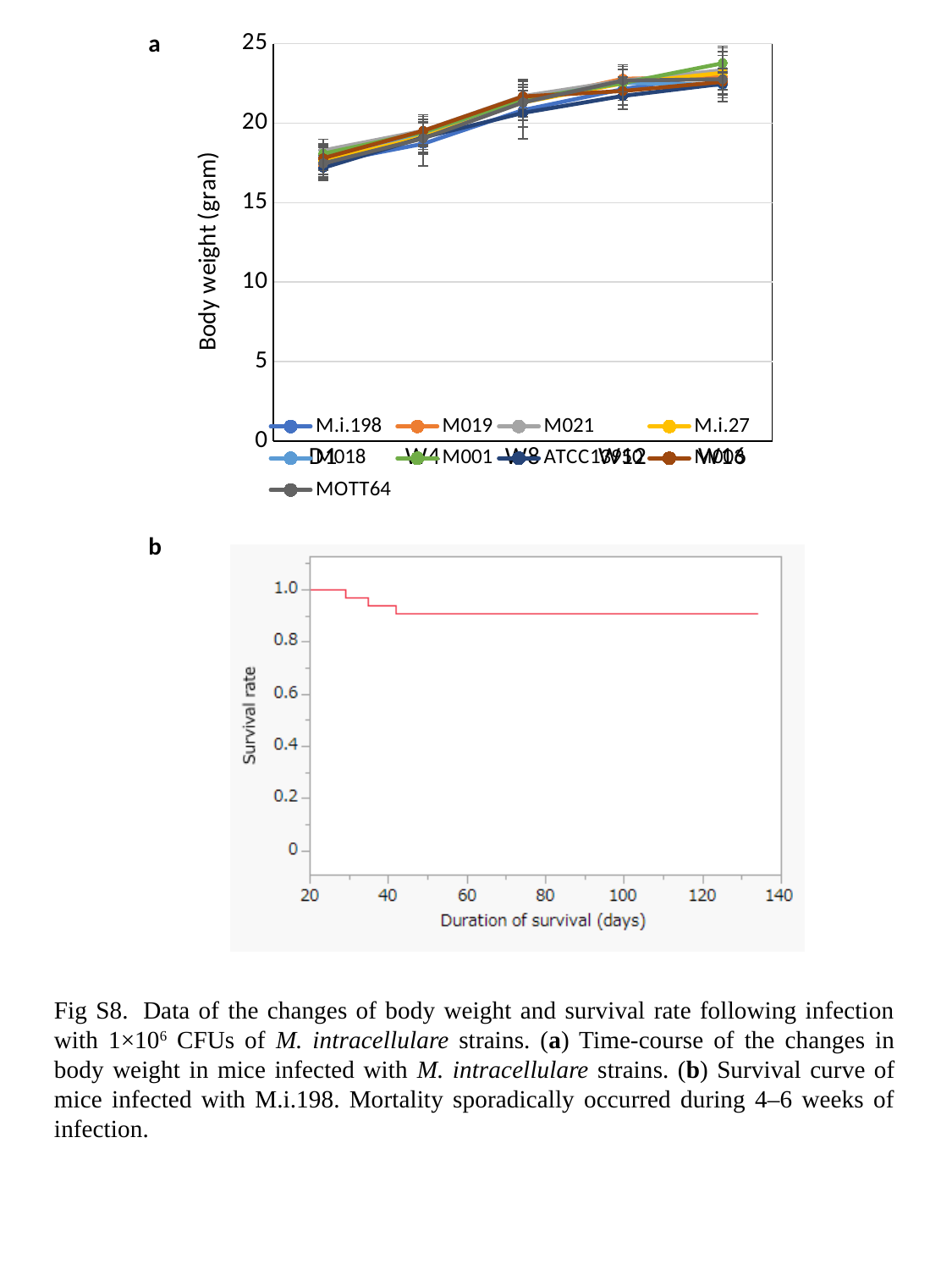

a
### Chart
| Category | M.i.198 | M019 | M021 | M.i.27 | M018 | M001 | ATCC13950 | M003 | MOTT64 |
|---|---|---|---|---|---|---|---|---|---|
| D1 | 17.48148148148148 | 17.310714285714287 | 18.28888888888889 | 17.614285714285714 | 17.882142857142856 | 18.08214285714286 | 17.192857142857143 | 17.80487804878049 | 17.471428571428568 |
| W4 | 18.704347826086952 | 19.38181818181818 | 19.51904761904762 | 19.190476190476186 | 19.41363636363636 | 19.425000000000004 | 19.099999999999998 | 19.51818181818182 | 19.040909090909093 |
| W8 | 20.823529411764707 | 21.4 | 21.7 | 21.30666666666667 | 21.4875 | 21.5 | 20.64666666666667 | 21.67777777777778 | 21.3 |
| W12 | 22.13636363636364 | 22.790000000000003 | 22.66666666666667 | 22.5 | 22.54 | 22.520000000000003 | 21.711111111111112 | 22.022222222222226 | 22.677777777777774 |
| W16 | 23.172727272727272 | 22.93 | 23.33333333333333 | 23.166666666666664 | 22.4 | 23.77 | 22.477777777777774 | 22.599999999999994 | 22.766666666666666 |b
Fig S8.  Data of the changes of body weight and survival rate following infection with 1×10^6^ CFUs of M. intracellulare strains. (a) Time-course of the changes in body weight in mice infected with M. intracellulare strains. (b) Survival curve of mice infected with M.i.198. Mortality sporadically occurred during 4–6 weeks of infection.
