# Supplementary material for: Virulence of Mycobacterium intracellulare clinical strains in a mouse model of lung infection – role of neutrophilic inflammation in disease severity
Source: BMC Microbiol. 2023 Apr 3;23:94. doi: 10.1186/s12866-023-02831-y (PMC10069106; doi:10.1186/s12866-023-02831-y)
Supplement: Supplementary file 12 — Additional file 12: Fig. S9. Data of the changes of body weight following infection with 2×10^7^ CFUs of M. intracellulare strains. a Time-course of the changes in body weight in mice infected with M. intracellulare strains. There was a significant difference in body weight in M.i.198-infected mice at 4 weeks of infection compared with mice infected with M.i.27, M018, M019 and M021 and the no-infection group. The body weight of mice infected with M019 was lower than that of mice infected with the intermediate virulence strains (M.i.27, M018) after 4 weeks of infection. The body weight of mice infected with M019 was lower than that of mice infected with M021 at 16 weeks of infection. The body weight of mice infected with M021 was lower than that of mice infected with the intermediate strains after 8 weeks of infection. Data on body weight was limited to surviving mice at the time-points of measurement. b The number of mice assayed for the measurement of body weight at each time-point. * Twenty mice were prepared at day 0 of the experiment, but two mice died the day after intratracheal instillation. [file 12866_2023_2831_MOESM12_ESM.pptx]

## Slide 1
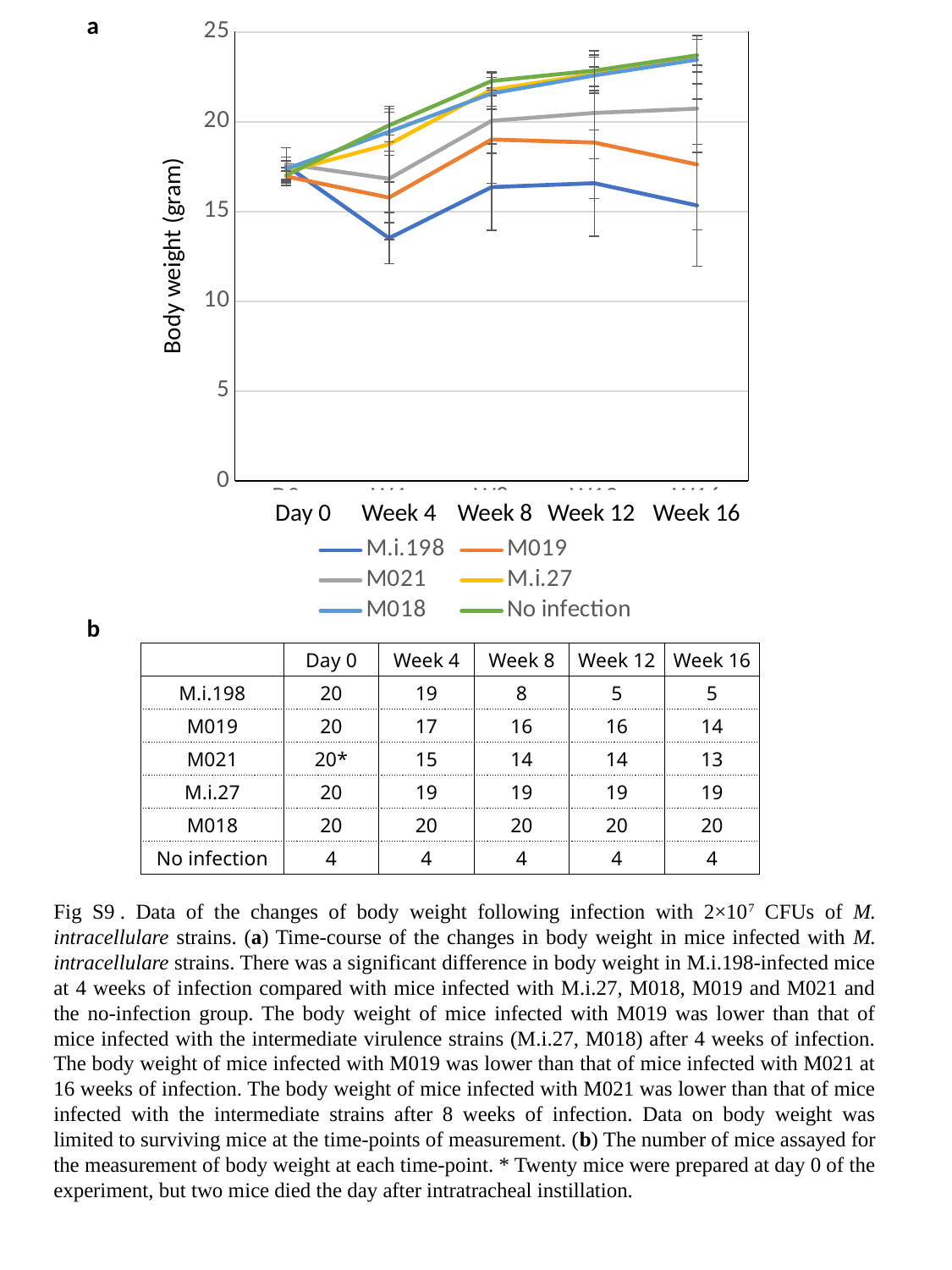

a
### Chart
| Category | M.i.198 | M019 | M021 | M.i.27 | M018 | No infection |
|---|---|---|---|---|---|---|
| D0 | 17.56 | 16.955 | 17.645000000000003 | 17.23 | 17.37 | 17.025 |
| W4 | 13.515789473684213 | 15.776470588235297 | 16.826666666666668 | 18.74736842105263 | 19.445 | 19.8 |
| W8 | 16.3625 | 19.0125 | 20.057142857142853 | 21.789473684210527 | 21.584999999999997 | 22.275 |
| W12 | 16.580000000000002 | 18.843749999999993 | 20.49285714285714 | 22.663157894736845 | 22.584999999999994 | 22.85 |
| W16 | 15.34 | 17.621428571428574 | 20.73076923076923 | 23.689473684210526 | 23.46 | 23.7 |Body weight (gram)
Day 0
Week 4
Week 8
Week 12
Week 16
b
| | Day 0 | Week 4 | Week 8 | Week 12 | Week 16 |
| --- | --- | --- | --- | --- | --- |
| M.i.198 | 20 | 19 | 8 | 5 | 5 |
| M019 | 20 | 17 | 16 | 16 | 14 |
| M021 | 20\* | 15 | 14 | 14 | 13 |
| M.i.27 | 20 | 19 | 19 | 19 | 19 |
| M018 | 20 | 20 | 20 | 20 | 20 |
| No infection | 4 | 4 | 4 | 4 | 4 |
Fig S9 . Data of the changes of body weight following infection with 2×10^7^ CFUs of M. intracellulare strains. (a) Time-course of the changes in body weight in mice infected with M. intracellulare strains. There was a significant difference in body weight in M.i.198-infected mice at 4 weeks of infection compared with mice infected with M.i.27, M018, M019 and M021 and the no-infection group. The body weight of mice infected with M019 was lower than that of mice infected with the intermediate virulence strains (M.i.27, M018) after 4 weeks of infection. The body weight of mice infected with M019 was lower than that of mice infected with M021 at 16 weeks of infection. The body weight of mice infected with M021 was lower than that of mice infected with the intermediate strains after 8 weeks of infection. Data on body weight was limited to surviving mice at the time-points of measurement. (b) The number of mice assayed for the measurement of body weight at each time-point. * Twenty mice were prepared at day 0 of the experiment, but two mice died the day after intratracheal instillation.
