# Supplementary material for: Virulence of Mycobacterium intracellulare clinical strains in a mouse model of lung infection – role of neutrophilic inflammation in disease severity
Source: BMC Microbiol. 2023 Apr 3;23:94. doi: 10.1186/s12866-023-02831-y (PMC10069106; doi:10.1186/s12866-023-02831-y)
Supplement: Supplementary file 13 — Additional file 13: Fig. S10. BALF and histological findings on the chemotherapeutic experiment. a Comparison of the infiltration of macrophages/monocytes (Right) and lymphocytes (Left) in the BALF before and after chemotherapy. *: significantly lower compared with all other groups and before chemotherapy, #: significantly higher compared with CAM monotherapy, RFP+EB+CAM (R+E+C), RFP+EB+CAM+AMK (R+E+C+A), and before chemotherapy. †: significantly higher compared with CAM monotherapy, R+E+C, and R+E+C+A. ‡: significantly higher compared with R+E+C+A. b Histological images of the lungs of M019-infected mice for each treatment group. Bars indicate 100 μm. [file 12866_2023_2831_MOESM13_ESM.pptx]

## Slide 1
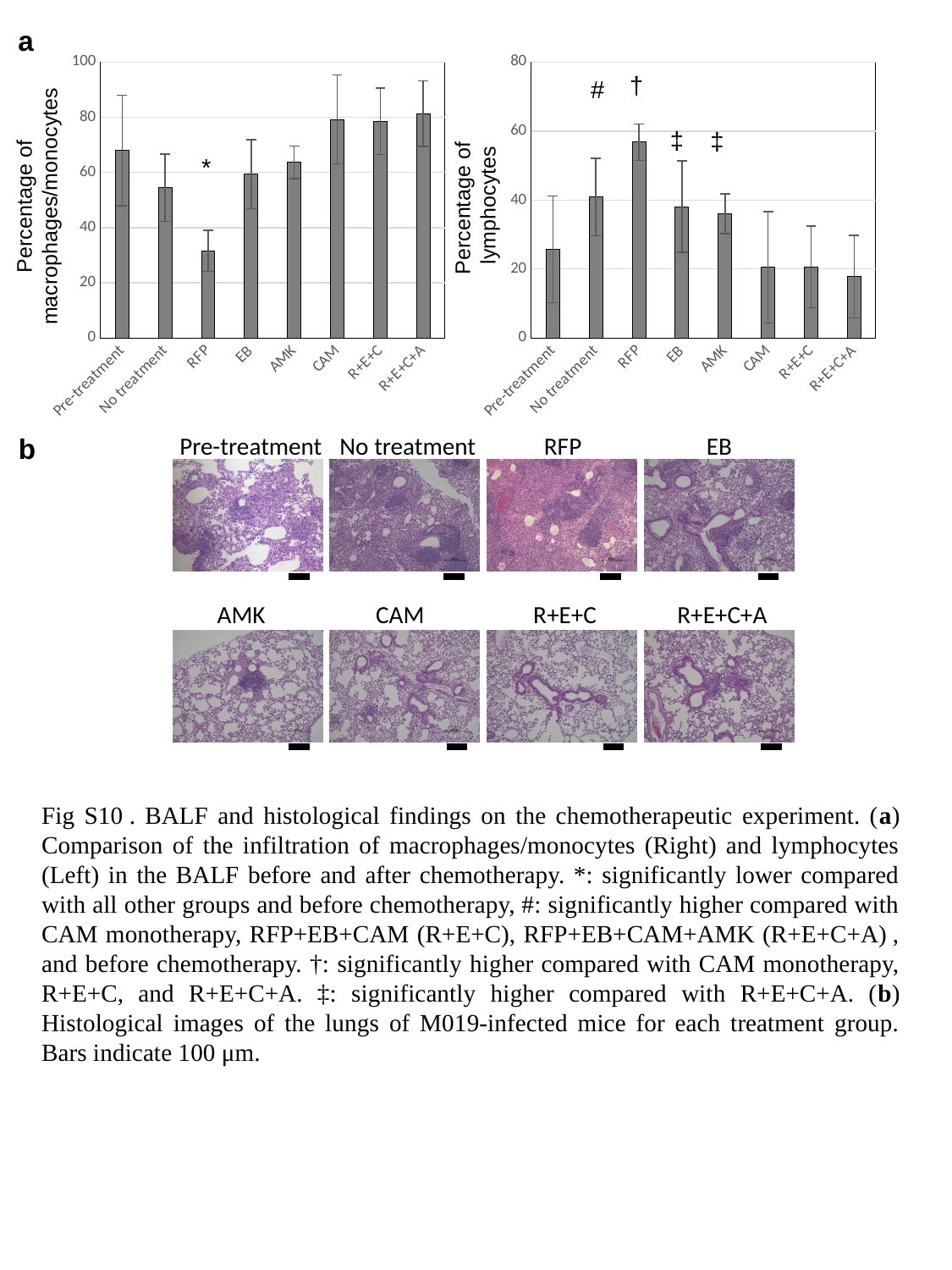

a
### Chart
| Category | |
|---|---|
| Pre-treatment | 68.0 |
| No treatment | 54.5 |
| RFP | 31.6 |
| EB | 59.3 |
| AMK | 63.7 |
| CAM | 79.2 |
| R+E+C | 78.5 |
| R+E+C+A | 81.3 |
### Chart
| Category | |
|---|---|
| Pre-treatment | 25.7 |
| No treatment | 40.9 |
| RFP | 56.8 |
| EB | 38.1 |
| AMK | 36.0 |
| CAM | 20.5 |
| R+E+C | 20.6 |
| R+E+C+A | 17.8 |†
#
‡
‡
*
Percentage of lymphocytes
Percentage of macrophages/monocytes
Pre-treatment
No treatment
RFP
EB
b
AMK
CAM
R+E+C
R+E+C+A
Fig S10 . BALF and histological findings on the chemotherapeutic experiment. (a) Comparison of the infiltration of macrophages/monocytes (Right) and lymphocytes (Left) in the BALF before and after chemotherapy. *: significantly lower compared with all other groups and before chemotherapy, #: significantly higher compared with CAM monotherapy, RFP+EB+CAM (R+E+C), RFP+EB+CAM+AMK (R+E+C+A) , and before chemotherapy. †: significantly higher compared with CAM monotherapy, R+E+C, and R+E+C+A. ‡: significantly higher compared with R+E+C+A. (b) Histological images of the lungs of M019-infected mice for each treatment group. Bars indicate 100 μm.
