# Supplementary material for: Virulence of Mycobacterium intracellulare clinical strains in a mouse model of lung infection – role of neutrophilic inflammation in disease severity
Source: BMC Microbiol. 2023 Apr 3;23:94. doi: 10.1186/s12866-023-02831-y (PMC10069106; doi:10.1186/s12866-023-02831-y)
Supplement: Supplementary file 14 — Additional file 14: Table S3. Bronchoalveolar lavage fluid (BALF) counts in mice infected with M019 and treated with chemotherapy. [file 12866_2023_2831_MOESM14_ESM.pptx]

## Slide 1
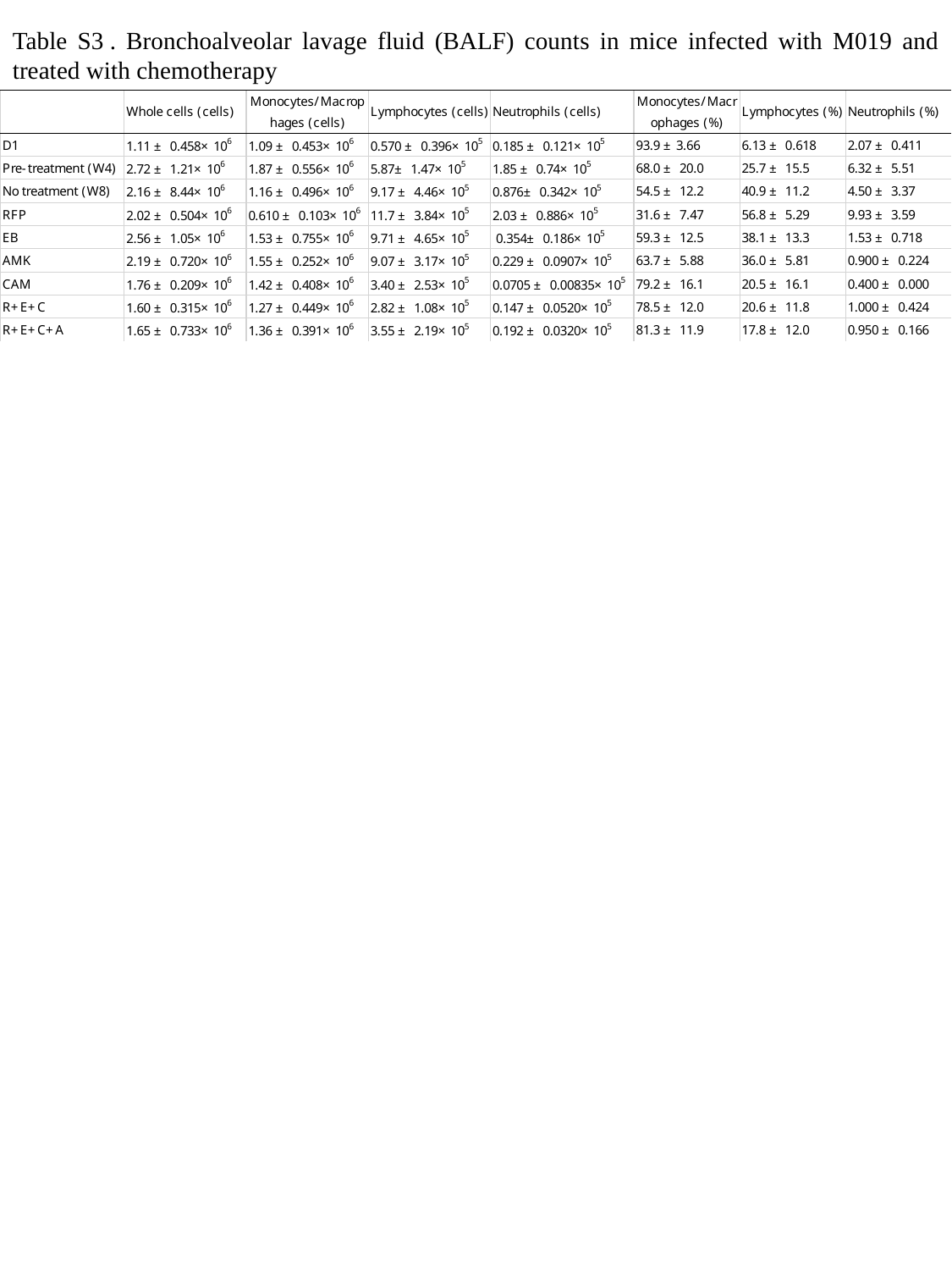

Table S3 . Bronchoalveolar lavage fluid (BALF) counts in mice infected with M019 and treated with chemotherapy
